# Whole Body Vibration Treatments in Postmenopausal Women Can Improve Bone Mineral Density: Results of a Stimulus Focussed Meta-Analysis

**DOI:** 10.1371/journal.pone.0166774

**Published:** 2016-12-01

**Authors:** Antonio Fratini, Tecla Bonci, Anthony M. J. Bull

**Affiliations:** 1 School of Life and Health Sciences, Aston University, Birmingham, United Kingdom; 2 Department of Bioengineering, Imperial College London, London, United Kingdom; Harvard Medical School/BIDMC, UNITED STATES

## Abstract

Whole body vibration treatment is a non-pharmacological intervention intended to stimulate muscular response and increase bone mineral density, particularly for postmenopausal women. The literature related to this topic is controversial, heterogeneous, and unclear despite the prospect of a major clinical effect.The aim of this study was to identify and systematically review the literature to assess the effect of whole body vibration treatments on bone mineral density (BMD) in postmenopausal women with a specific focus on the experimental factors that influence the stimulus. Nine studies fulfilled the inclusion criteria, including 527 postmenopausal women and different vibration delivery designs. Cumulative dose, amplitudes and frequency of treatments as well as subject posture during treatment vary widely among studies. Some of the studies included an associated exercise training regime. Both randomized and controlled clinical trials were included. Whole body vibration was shown to produce significant BMD improvements on the hip and spine when compared to no intervention. Conversely, treatment associated with exercise training resulted in negligible outcomes when compared to exercise training or to placebo. Moreover, side-alternating platforms were more effective in improving BMD values than synchronous platforms and mechanical oscillations of magnitude higher than 3 g and/or frequency lower than 25 Hz were also found to be effective. Treatments with a cumulative dose over 1000 minutes in the follow-up period were correlated to positive outcomes.Our conclusion is that whole body vibration treatments in elderly women can reduce BMD decline.However, many factors (e.g., amplitude, frequency and subject posture) affect the capacity of the vibrations to propagate to the target site; the adequate level of stimulation required to produce these effects has not yet been defined. Further biomechanical analyses to predict the propagation of the vibration waves along the body and assess the stimulation levels are required.

## Introduction

Musculoskeletal pathologies and age-related decline of muscles, bones and joint function represent the primary contributors to loss of quality of life in ageing [1]. Chronic conditions such as osteopenia and osteoporosis pose serious challenges to public health management [2], because of the expected rise in numbers of the elderly in the European Union [3].

Osteopenia and osteoporosis are systemic skeletal disorders characterised by low bone mass and micro-architectural deterioration of bone tissues, which contribute to the increase in bone fragility and its susceptibility to fracture. Osteoporotic fractures commonly occur at the spine, hip, distal forearm and proximal humerus [4].

The most effective ways to prevent or delay the effect of such musculoskeletal disorders involve pharmaceutical intervention with or without physical activity [5]. The evidence suggests that uptake of increased physical exercise to mitigate such musculoskeletal conditions in the elderly is low [6].

Whole body vibration (WBV) treatment, which uses mechanical stimulation delivered via vibrating platforms, has emerged as a potential alternative for muscle and bone stimulation. Literature reports of physiological adaptation to vibratory mechanical loads thus proposed a novel non-pharmacological approach to the treatment of musculoskeletal disorders [7] and many authors have investigated the effect of WBV on bone mineral density (BMD) with a wide range of outcomes [8, 9].

WBV is a stimulus that involves the combination of various mechanical variables. Vibrations are transmitted through the kinematic chain of the body; the combination of frequency, amplitude of the stimulus, subject posture and vibration delivery design can dramatically change the actual stimulus at the target site [10, 11].

Thus, the lack of understanding of the propagation of WBVs along the body, as well as the estimation of local stimulus at the target site, may prevent the appropriate design of treatments while also reducing their effectiveness.

The aim of this study was to identify, systematically review and assess the literature on the effect of WBV on bone mineral density in postmenopausal women, with a particular focus on the factors that influence the stimulus characteristics as well as its transmissibility. To our knowledge, this is the first systematic assessment relating vibration delivery design, magnitude, frequency, subject’s posture or simultaneous exercise, follow-up period and cumulative dose with the treatment outcomes at the target site.

## Methods

This systematic review and meta-analysis was conducted in accordance with the procedures developed by the Cochrane Collaboration [12] and the Preferred Reporting Items for Systematic reviews and Meta-Analyses (PRISMA) guidelines [13]. Further details in S1 and S2 Files. The search strategy was defined a priori: this study was aimed at understanding the influence of WBV treatments in leading to better BMD outcomes in postmenopausal women. Specifically, vibratory treatments were compared to exercise training or absence of interventions. However, since variations in magnitude [14], vibration delivery design and frequency [11], subject’s posture [15, 16] and other variables modify the actual stimulus at the target muscle or bone, subgroup analyses were performed. The influence of each of these variables on the BMD values was assessed first; thereafter their combinations for specific anatomical areas were analysed and reported.

### a. Data Sources

Six electronic databases were searched starting from the earliest date using the following keywords: *whole body vibration*, *vibrating*, *frequency*, *bone mineral density*, *bone density*, *postmenopausal*. The databases used were: MEDLINE, Cochrane Library, IEEE Xplore, Scopus, and Web of Knowledge. As an example of full search, the string used for the Scopus database is here reported: *“(TITLE-ABS-KEY (whole body vibration)) AND (TITLE-ABS-KEY (bone mineral density) OR TITLE-ABS-KEY (bone density)) AND (LIMIT-TO (DOCTYPE*, *“ar”)) AND (LIMIT-TO (LANGUAGE*, *“English”))”*.

Unpublished trials were searched using clinical trials registries (http://ClinicalTrials.gov and http://Controlled-trials.com). A hand search of reference lists of the retrieved papers was additionally completed. The electronic sources were last searched on 4^th^ December 2015.

### b. Study Selection

Studies that examined the effect of WBV on BMD in postmenopausal women were selected. Included articles comprised randomized controlled trials (RCTs) and controlled clinical trials (CCTs). Interventions accepted were WBV (via vibrating platforms) either with or without combined exercise training. Eligible control groups included traditional exercise training or light/absent physical activity. No restrictions on vibration delivery design, frequency, magnitude and cumulative dose (total stimulus delivered to the patients) were considered.

The exclusion criteria were: research studies in patients with primary diagnosis of specific pathological conditions (e.g. stroke), use of medications (e.g. alendronate), studies on male subjects, athletes or exercise trained patients, and studies published in books or conference proceedings. Studies following an intention-to-treat (ITT) approach were also excluded [17, 18]. To avoid articles exclusions, WBV reviewes and other meta-analysis articles were also investigated.

### c. Data Extraction

Data were extracted from each of the selected studies. The following information was recorded: generic paper descriptions (authors, title and year), research design, participant characteristics, stimulation details (i.e. delivery design, magnitude, frequency, and posture during vibratory stimulation), and BMD details. Corresponding authors were contacted in case of doubt or unrecoverable data.

#### Outcomes Evaluation

In osteopenic and osteoporotic patients BMD values decrease with time. Hence, a treatment addressing osteopenia or osteoporosis is meant to reduce, stop or even reverse that decline. Therefore, BMD values of the selected sites were reported for each study both pre and post WBV treatment, as mean and standard deviation (SD). BMD values were reported in g/cm^2^ as quantified by dual energy X-ray absorptiometry [19].

Whole body, hip and lumbar spine areas are typically investigated due to their importance in the assessment of the osteoporosis; BMDs of the following anatomical sites were therefore considered: Lumbar Spine (LS), Total Hip (TH), Femoral neck (FN), Trochanter (TR), and whole body (WB). When BMD data of the TH site were available, these values were preferred to the combination of FN and TR [20].

Different subgroup analyses were performed focusing on applied vibration (frequencies, magnitudes and delivery design), exercise or static posture on the platform, cumulative dose, follow-up period, and on specific anatomical sites. Vibration frequencies were classified as low or high (below or above 25Hz), to take into account their different transmissibility at the level of the hip, as it is known that in commonly used treatment postures (standing or squat) stimuli above 25 Hz are strongly damped [15].

#### Estimations and Statistical Inferences

Where data were not reported in the studies or could not be obtained directly from the authors, estimations or statistical inferences were used. The mean absolute change in BMD value, for each of the treatment and control groups, was obtained as the difference of the post-intervention and the baseline mean. The SD value relative to the mean absolute change in BMD values was computed using Follmann’s method [12]. The correlation coefficient used (0.98) was estimated by averaging *r* values obtained for different anatomical sites (FN, TR, LS, TH and WB) from the selected studies. Statistical manipulations were also used to convert confidence intervals (CI) [21] or standard errors [22–24] to SDs.

If not described, the maximal acceleration at the level of the platform (*a*_*max*_, vibration intensity or magnitude) was estimated using the following formula:

$$|a_{max}|=\frac{A∙{\left(2\pi f\right)}^{2}}{g}$$ (1)

Where *A* is the amplitude of the platform oscillations (half of the peak to peak value), *g* is the gravitational acceleration (9.81 m/s^2^ was used) and *f* is the vibration frequency. As can be noted from Eq 1, the acceleration depends linearly on the amplitude of the oscillation itself and quadratically on the square of the pulsation (*ω* = 2*πf*). This assumes that the oscillation is sinusoidal, an assumption that is justified from the literature for vibrating platforms [25].

Cumulative dose, i.e. the extent of vibration (in min) to which a subject is exposed during the follow-up period, was also estimated. In case of generic information, such as *x months of treatment and y session per week*, a value of 4.3 weeks per month was used.

All analyses were performed using OpenMeta[Analyst] software [26], 95% confidence intervals (CIs) were assessed for all the relevant BMD measures. I^2^ statistics was performed to quantify heterogeneity. The random-effects model was used to pool outcomes as it is identical to the fixed-effect model if no statistical heterogeneity (i.e. I^2^ = 0%) is present [27].

When the meta-analyses suggested an overall treatment effectiveness, the resulting weighted mean difference (WMD) [12], was reported as a percentage of the baseline BMD values of the selected studies.

**Fig 1:**
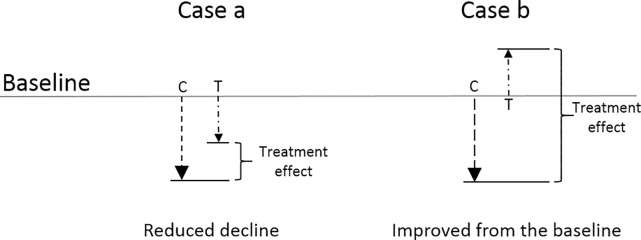
Examples of WBV potential effects on BMD values. The arrows describe the change with respect to the baseline BMD values between control (C) and treatment (T).

The design of these meta-analyses does not distinguish between a reduction in BMD decline (Case a in Fig 1) or an increase in BMD values from the baseline (Case b in Fig 1).

In order to provide a clearer comparison of the BMD improvement in treatment and control groups, the difference in BMD change, defined as Δ*BMD* = |*BMD*_*post*_ − *BMD*_*pre*_|, was used to calculate the relative effect of treatment using:

$$RE=\frac{{\Delta BMD}_{T}-{\Delta BMD}_{C}}{{\Delta BMD}_{c}}∙100$$ (2)

Where *RE* is the relative effect of the treatment with respect to the control group, Δ*BMD*_*T*_ is the weighted absolute mean change for treatment groups and, Δ*BMD*_*C*_ is the weighted absolute mean change for control groups. RE describes the relative difference existing between treatment and control group outcomes with respect to the control group outcomes. RE is negative when the treatment has the overall effect of reducing the BMD decline (Case a, Fig 1) and it is positive when the treatment can reverse the BMD decline and baseline values are lower than the end of treatment ones (Case b, Fig 1).

## Results

**Fig 2:**
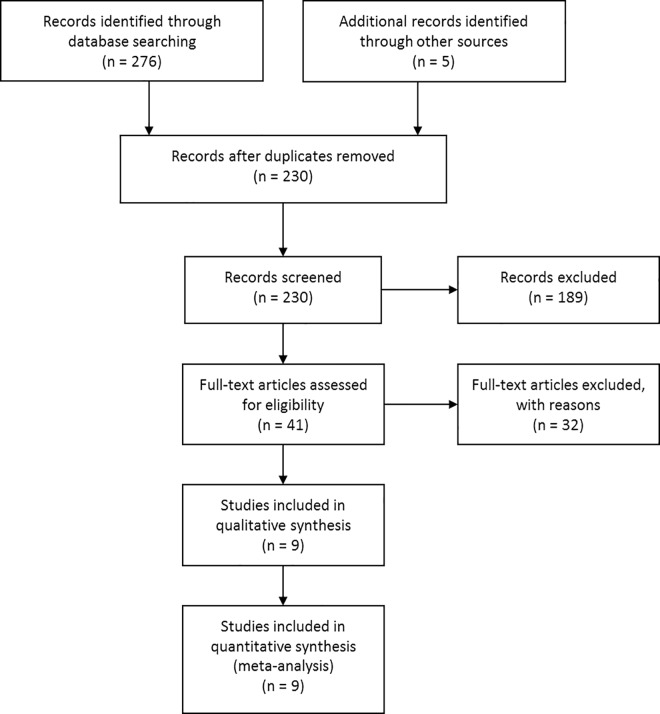
Flow of study selection.

358 potentially relevant references were retrieved of which 128 were duplicates. A further 189 papers were excluded due to non-eligible population, absence of control group, and use of medications (Fig 2). Nine studies met the eligibility criteria [21–24, 28–32], three were CCTs [21, 23, 28] while the others were RCTs. The number of included trials was insufficient to shape a funnel plot or to use more advanced regression-based estimations; publication bias could not be properly assessed.

Full trial characteristics are summarised in Table 1. Studies differ in participant numbers (range 28–135), age (range 46–93 years), follow-up period (range 6–18 months), type of device/stimulus (side-alternating rotation and synchronous vertical tilting), or amplitudes (0.3-18g) and frequency of the stimulus (12.5-40Hz), static posture vs exercise on platform and cumulative dose of treatment (38–2160 min). Control groups include no activity vs exercise training.

**Table 1:** Characteristic of the selected articles.

**Source**	**Subjects**	**T-score [Mean ± SD or range]**	**Supplements**	**Age in years [Mean ± SD or range]**	**No. of Subjects**	**Treatment Type**	**Control**	**Follow up period [months]**	**BMD [g/cm**^**2**^**]**	**Vibration frequency [Hz]**	**Vibration magnitude at platform level [g]**	**Vibration device/type**	**Cumulative dose [min]**	**Subject Posture**	**Study type**
**Beck et al. ^22^, 2010**	Postmenopausal women (>5yrs)	-2.2 ± 1.1 (-4.18,-0.6)	NO	71 ± 9	47	WBV	• Placebo	8	• Femoral neck • Trochanter • Lumbar spine • Whole body • Proximal forearm	• 30 • 12.5	• 0.3 • 1	• Juvent 1000/ synchronous • Galileo 2000/side-alternating	• 1032* • <412*	• S • SKF	RCT
**Bemben et al. ^23^, 2010**	Postmenopausal women (>5yrs)	T-score > - 2.5	NO	55–71	55	WBV+Resistance training	• Placebo • Resistance training	8	• Femoral neck • Trochanter • Lumbar spine • Whole body • Total hip • Radius 33%	30–40	2.16 to 2.8	Power plate/synchronous	38.25	• SE + E • S + E	CCT
**Gusi et al. ^21^, 2006**	Postmenopausal women (>5yrs)	NA	NA	66 ± 5	28	WBV	• Placebo (walking)	8	• Femoral neck • Trochanter • Ward’s triangle • Lumbar spine	12.6	0.96	Galileo 2000/side-alternating	516	SKF 60°	CCT
**Karakiriou et al. ^24^, 2012**	Early postmenopausal women (1.80–6.64 yrs)ˆ	T-score < - 2	NO	46–62	33	WBV	• Placebo • Aerobic and resistance training	6	Lumbar spine (L2–L4)	35–40	3.7 to 4.8**	Nemes LCB/side-alternating	• 474* • 271*	• SKF • BOL	RCT
**Lai et al. ^30^, 2013**	Postmenopausal women (0–30 yrs)	92.5% T-score<-1.6	NO	46–69	28	WBV	• Placebo	6	Lumbar spine (L1–L4)	30	3.2	LV-1000; X-trend Fitness/synchronous	387	S	RCT
**Ruan et al. ^28^, 2008**	Postmenopausal women (0–30 yrs) ˆ	T-score < -2.5	NO	61 ±8 Treatment64 ± 5 Control	94	WBV	• Placebo	6	• Femoral neck • Lumbar spine(L2-L4)	30	18**	ZD 10 Beijing Maidakang Med/ synchronous	1290	S	CCT
**Santin-Medeiros et al. ^31^, 2015**	Postmenopausal women (ND)	45.3% T-score<-1.6	NA	71–93	37	WBV	• Placebo	8	• Total hip • Femoral neck • Trochanter • Intertrochanter • Ward’s area	20	3.22 **	Fitvibe Excel Pro, Bilzen,Belgium/vertical	130.5	E	RCT
**Verschueren et al. ^29^, 2004**	Postmenopausal women (ND)	T-score > - 2.5	NO	58–74	70	WBV+Resistance training	• Placebo • Resistance training	6	• Lumbar spine • Total hip • Whole body	35–40	2.28 to 5.09	Power plate/ synchronous	< 2160	E	RCT
**von. Stengel et al. ^32^, 2011**	Postmenopausal women (ND)	NA	Vitamin D and calcium (>400, 1500 mg/day)	65–76	135	WBV+Aerobic+resistance training	• Placebo • Aerobic and resistance training	18	• Lumbar spine (L1-L4) • Total hip	25–35	4.3 to 8.4**	Vibrafit, Solms/ side-alternating	1858*	E	RCT

* A value of 4.3 weeks per month was used to estimate the cumulative dose

** Estimated using the reported data for frequency and amplitude of the vibration

ˆ Estimated using the reported data for years since menopause

NA: not available–ND: not declared

S: Subject stands on the platform in full extension

SE: Subject is seated on the platform

SKF α: Subject stands on the platform with knee flexion at angle α (when available)

BOL: Balanced on one leg, leaning on a handlebar

E: Exercises on the platform

RCT/CCT: randomized controlled trial and controlled clinical trial

### a. Cumulative Effects Of Whole Body Vibrations Treatment

To assess the effect of WBV treatments, a cumulative meta-analysis was performed: treatment groups (WBV and WBV combined with exercise) were compared with control groups (exercise training and placebo) for all the available anatomical sites. The results showed a high heterogeneity (I^2^ = 94%, p<0.001) and a weighted mean difference equal to zero (p = 0.812). This suggested that the effect of each specific variables should be investigated separately (e.g. magnitude and frequency of the vibration, vibration delivery design, etc.). Analyses performed and their results are reported in the following paragraphs.

### b. Effect Of Magnitude

**Fig 3:**
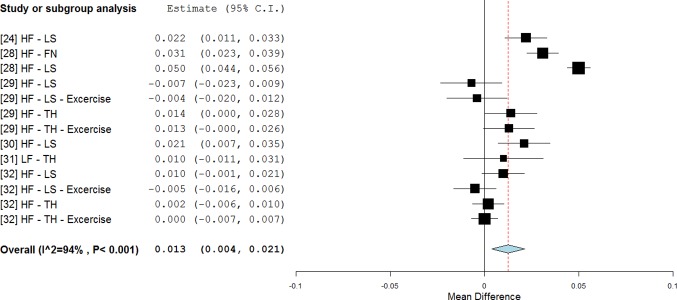
Subgroup analysis of whole body vibration effect on BMD outcomes of the available anatomical sites when the subjects were stimulated with a vibration magnitude higher than 3 g. The forest plot shows the weighted mean difference between the control and the whole body vibration group for the selected studies.

The magnitude of the acceleration produced at the platform level is important for the delivery of the stimulus: higher magnitude stimuli can overcome the damping effect produced by soft tissues and may effectively reach the target sites. The analysed studies include stimuli varying from 0.3 g [22] to 18 g [28]. Our analyses showed that if the magnitude delivered was greater than 3 g [24, 28–32] there was a significant effect of treatment (0.013 g/cm^2^, p = 0.005, WMD = 1.5%) with respect to the relevant control groups (Fig 3). BMD decline can be potentially reversed (RE = 67%).

### c. Effect Of Frequency

**Fig 4:**
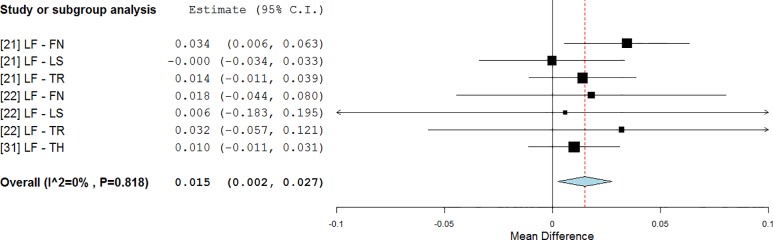
Subgroup analysis of whole body vibration effect on BMD outcomes of the available anatomical sites when the subjects were stimulated using low frequency vibrations. The forest plot shows the weighted mean difference between the control and the whole body vibration group for the selected studies.

Frequencies used in WBV vary widely among the treatments: from 12.5 Hz [22] to 40 Hz [23, 29]. By using the frequency classification previously described, a significant overall improvement (0.015 g/cm^2^, p = 0.019, Fig 4) was found for studies that utilise LF WBV (from 12.5 Hz to 20 Hz) [21, 22, 31]. The BMD decline was lower than in the control groups (RE = -29%, WMD = 2.0%).

### d. Effect Of Vibration Delivery Design

WBV treatments are mainly based on two delivery designs (see Table 1). As well described in [25],vibrating platforms can generate “*reciprocating vertical displacements on the left and right side of a fulcrum*, *or the whole plate can oscillate uniformly up and down”*. These are referred to as side-alternating and synchronous platforms, respectively [33].

**Fig 5:**
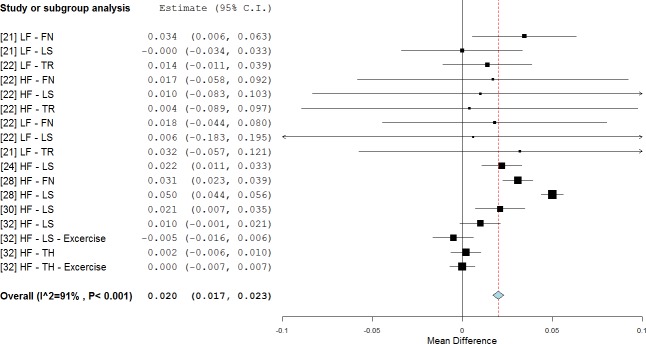
Subgroup analysis of whole body vibration effect on BMD outcomes when the stimulus was delivered using side-alternating platforms. The forest plot shows the weighted mean difference between the control and the whole body vibration group for the selected studies.

The analyses showed a significant overall improvement (0.020 g/cm^2^, p<0.001, WMD = 2.5%) in subjects treated with side-alternating platforms (Fig 5) [21, 22, 24, 28, 30, 32]. BMD values were higher than the baseline (RE = 73%). No improvement was found for tilting platforms.

### e. Effect Of Posture

Posture affects the transmission of the stimulus along the body. This is emphasised if the subject continuously changes pose during the treatment, as happens when exercising on the platform.

**Fig 6:**
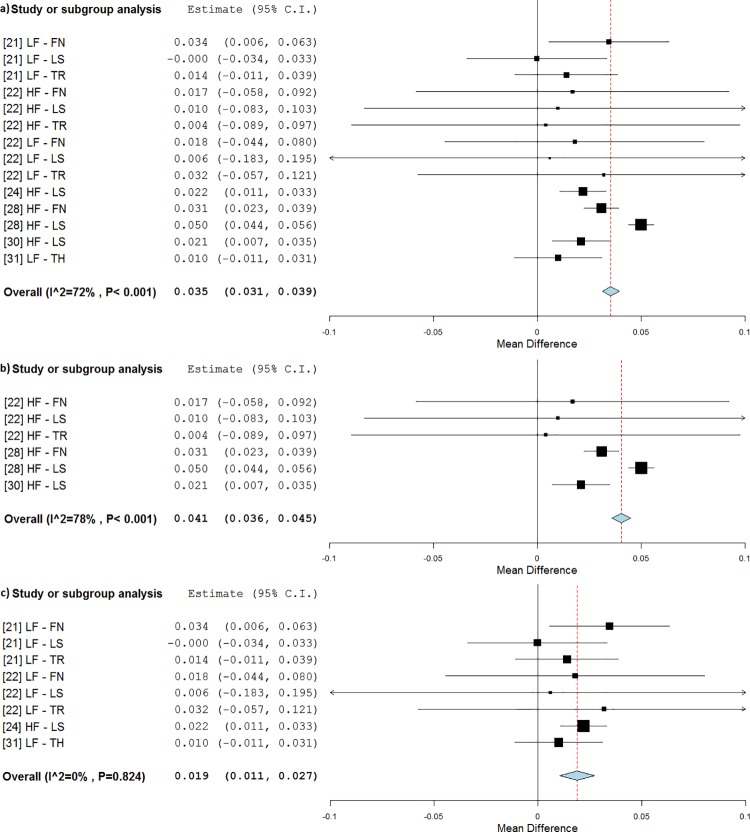
Subgroup analysis of whole body vibration effect on BMD outcomes of the available anatomical sites when the subjects hold a generic static posture on the platform (a), a full standing posture (b) and hack squat posture (c). The forest plots show the weighted mean difference between the control and the whole body vibration group for the relevant studies.

In three studies, exercises were performed during WBV treatments [23, 29, 32]. Two of these also incorporated a standard training program together with the stimulations [23, 32]. No differences in BMD values were found with respect to either of the control groups (placebo or exercise training). Conversely, when WBV was not associated with simultaneous exercises [21, 22, 24, 28, 30, 31], a difference of 0.035 g/cm^2^ (p<0.001, WMD = 4.5%) was observed (Fig 6A). BMD values were higher than the baseline (RE = 42%).

Two static postures were generally found in the studies used: full standing and hack squat. While both postures seemed effective, BMD values were found to be higher than the baseline in full standing subjects (0.041 g/cm^2^, p<0.001, WMD = 5.5%, RE = 112%) (Fig 6B) [22, 28, 30], while the BMD decline was reduced for subject treated in hack squat (0.019 g/cm^2^, p<0.001, WMD = 2.4%, RE = -35%, Fig 6C) [21, 22, 24, 31].

### f. Cumulative Dose And Follow-Up Period

There is no direct correlation between the cumulative dose of vibration, measured in minutes, and the follow-up period (Table 1). Six [24, 28–30], eight [21–23, 31] or eighteen [32] months were found as follow-up periods in the selected studies.

**Fig 7:**
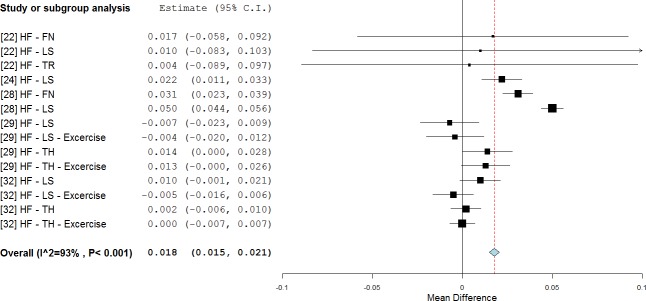
Subgroup analysis of whole body vibration effect on BMD outcomes observed when the cumulative dose of the treatment was delivered for more than 1000 minutes. The forest plot shows the weighted mean difference between the control and the whole body vibration group.

**Fig 8:**
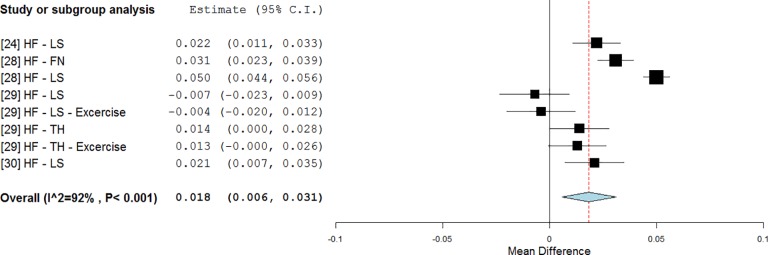
Subgroup analysis of whole body vibration effect on BMD outcomes observed when the treatment was delivered for six months. The forest plot shows the weighted mean difference between the control and the whole body vibration group.

High cumulative doses and short follow-up periods where found to be the most effective in WBV treatments. In detail, an overall positive effect on BMD outcomes (0.018 g/cm^2^, p < 0.001, WMD = 2.2%) was observed for those studies with a cumulative dose higher than 1000 min [22, 24, 28, 29, 32] (Fig 7), and also for studies with six months of follow-up (0.018 g/cm^2^, p = 0.002, WMD = 2.2%, Fig 8). Overall, BMD values after treatments were above the baseline in both treatment groups (RE = 75% and 42%, respectively).

### g. Effects Of WBV Variables On Different Anatomical Sites

Each of the analysed treatment variables has a specific effect on the BMD values. However, since these variables might also modify the stimulus locally at the target site, we have also explored their combination for the highlighted anatomical areas.

**Fig 9:**
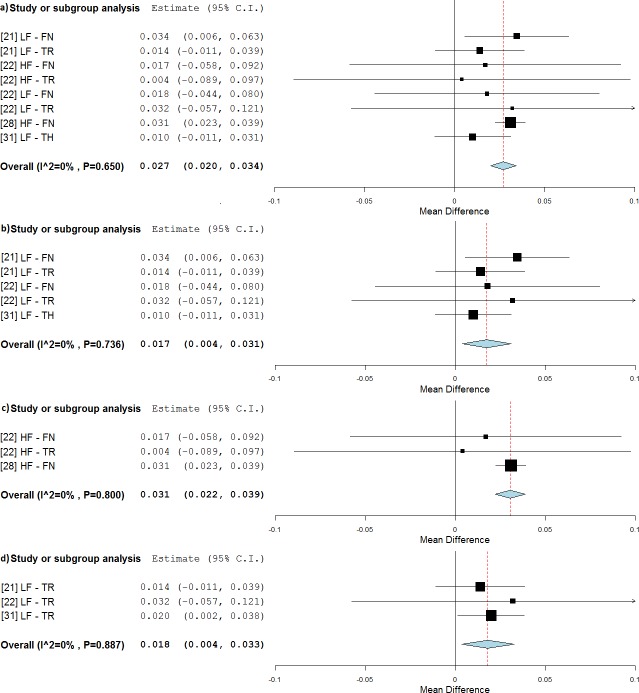
Subgroup analysis of whole body vibration effect on BMD outcomes observed on the hip site when treatments were not associated with simultaneous exercises. The forest plots show the weighted mean difference between the control and the whole body vibration group for the following subgroups: all frequencies (a), low frequencies (b), high frequencies (c), and TR site at low frequencies (d).

In detail, on the hip region a significant change in BMD was found for subjects treated in static postures (0.027 g/cm^2^, p <0.001, WMD = 3.9%, Fig 9A) [21, 22, 28, 31] and the BMD decline was almost avoided (RE = -4%). WBV treatments were effective for both LF (0.017 g/cm^2^, p = 0.011, WMD = 2.4%, Fig 9B) and HF stimulations (0.031 g/cm^2^, p<0.001, WMD = 4.7%, Fig 9C). It should be noted that the hack squat posture was found for the majority of the studies utilising LF during the stimulation, while for studies using HF the subjects were all treated in full standing. The BMD decline was lower with LF treatments (RE = -27%) and was reversed with HF treatments (RE = 17%). A further subgroup analysis (Fig 9D) revealed that LF treatments might specifically be targeting the TR area (0.018 g/cm^2^, p = 0.014, WMD = 3.0%, RE = -46%).

**Fig 10:**
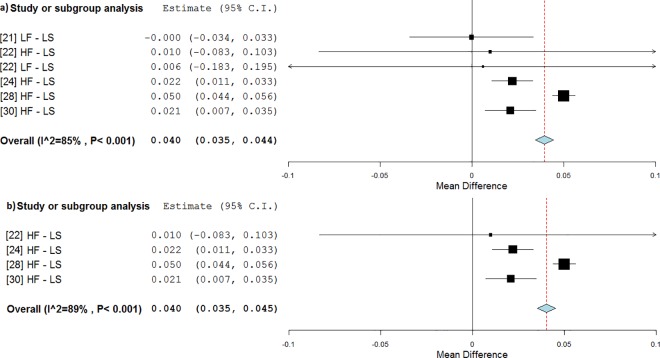
Subgroup analysis of whole body vibration effect on BMD outcomes observed at the lumbar spine site when treatments were delivered in standing postures. The forest plot shows the weighted mean difference between the control and the whole body vibration group for the following subgroups: all frequencies (a), high frequencies (b).

The lumbar spine area was found to be significantly influenced by WBV treatments in static postures (0.040 g/cm^2^, p<0.001, WMD = 4.6%, Fig 10A), reversing the BMD decline (RE = 131%). HF stimulation seemed to increase BMD values (0.040 g/cm^2^, p<0.001, WMD = 4.6%, RE = 178%, Fig 10B).

## Discussion

Two previous studies systematically analysed the effectiveness of vibratory stimulations, however, their conclusions are controversial. Slatkovska et al. [8] reported a small improvement in hip BMD while Lau et al. [9] concluded that vibratory stimulation has no overall effect on BMD amongst old women. The differences in study selection and data pooling methodology could have contributed to their discrepancy: in particular, the use of standardized mean difference in [9], which is generally used when studies measure the same outcome in different ways/scales [12], as well as the inclusion of the most recent studies, probably led to the non-significant results. In addition, although Slatkovska et al. [8] did not find intensity related changes, none of these studies dealt with frequency analyses, vibration delivery design transmissibility, and all the key parameters that have been highlighted in this article.

In this study, WBV stimulation was shown to produce a significant improvement (WMD ranged from 2 to 5.5%) in BMD values at the hip and spine when compared with placebo control groups. WBV treatments have shown the capacity to reduce and even reverse the decline of BMD due to osteopenia or osteoporosis.

However, vibration transmissibility issues could strongly influence the outcomes of a WBV treatment [11, 16]. The effectiveness of vibrations depends on a combination of variables. Mechanical oscillations are transmitted from the plate to the target muscle/bone, through the kinematic chain of the body, which varies with the pose that subjects hold on the platform. Moreover, as previously clarified, if the amplitude of the mechanical oscillation is kept constant, the intensity of the stimulus (i.e. the acceleration) applied to the body varies with the square of the pulsation. In turn, low vibration frequencies produce smaller stimuli compared to high frequencies. For example, a sinusoidal oscillation of 2 mm may generate an intensity of 1.8 *g* or 7.2 *g* at frequencies of 15 and 30 Hz, respectively. The role of the different variables set to deliver the stimulation was therefore investigated and some key results of this study can be highlighted.

Vibration delivery design has a strong impact on the outcomes: side-alternating platforms seemed the most effective. It is hypothesised that this is due to the similarity of the stimulus with gait.

Exercising on the platform during WBV treatments has shown not to affect BMD outcomes. Results are negligible when compared to exercise training or to placebo suggesting that the association of exercise with vibrations probably influences the transmission and action of the mechanical stimulus on the target site, especially if concomitant. These results are in agreement with the findings of the article by Harazin and Grzesik [15]: static postures should be used for treatments; these seemed effective for LS and TH areas. In full standing subjects, our analyses have shown better improvement of BMD values in either HF or LF stimulations; while in hack squat LF seemed to have a favourable effect especially for the TR site. It is worth noting that this is the only anatomical landmark reported on that is directly connected to muscles and it is hypothesised that this effect is due, in part, to muscle contractions that enable the person to maintain this posture.

Higher vibration magnitudes (≥3 *g*) produce better results [24, 28, 30, 31]; a more intense stimulus can overcome the damping effect of soft tissues and reach with adequate energy the target site (Figs 8 and 9). As the vibration effect is negligible on most of the upper body, BMD values for the whole body did not show noticeable changes. Mechanical stimuli delivered through the lower limbs using vibration platforms cannot reach with a sufficient intensity the muscles/bones of the upper body thus producing negative results for WB BMD values.

The time over which the subjects are exposed to the vibration, i.e. the cumulative dose, was also found to be positively correlated to the effectiveness of WBV treatments. It seemed to have a predominant role compared to the whole treatment time (follow-up period). Similar results can be found in the work of Gusi et al. [21] who highlighted the importance of the number of treatment sessions per week (which in turn modifies the cumulative dose) to obtain a significant effect on BMD values.

It is also worth noting that the skeleton’s sensitivity to WBV seems to be inversely related to the initial BMD values [8]. A greater effect could be observed if baseline values are lower [30]. Therefore, including normal, osteopenic or osteoporotic women in the sample consistently affects the results and their variability. Indeed, in our analyses the study with the widest CI was found to use the most heterogenious groups (-4.18 < T-score < -0.6) [22].

Finally, some limitations of this study need to be considered. Quantifying BMD by dual energy X-ray absorptiometry is dependent on the operator, machine used and machine calibration. The analysis of the differences (post-treatment minus pre-treatment) and the presence of a trained operator for most of the eligible studies, helped to reduce these errors. The number of RCTs or CCTs available was limited and the sample size of the considered trials is generally small, in addition, ITT analyses were not included for data extraction. These conditions may have biased the results to more favourable outcomes.

## Conclusions

Whole body vibrations can reduce the decline of bone density in postmenopausal women and can be potentially used to limit pathologies of ageing such as osteoporosis and sarcopenia. However, this study shows that WBV protocol design needs further analysis to tune the variables to achieve the most significant outcome.

Some authors argue that specific mechanical frequencies act on the piezoelectric properties of bones while others believe that the effect of vibration on bone tissue is subsequent to the renewed muscular tone induced by the stimulus itself. Our analysis showed that multiple factors have to be taken in to account when designing a treatment.

Biomechanical analyses as well as the use of simulation tools are therefore required to predict the propagation of the mechanical waves along the body. This in turn, could also lead to better understanding the extent of the stimulus applied while correlating it with the resultant effects at target sites.

## Supporting Information

S1 File PRISMA 2009 Flow Diagram (DOC)

S2 File PRISMA 2009 Checklist (DOC)
