# Supplementary material for: Whole Body Vibration Treatments in Postmenopausal Women Can Improve Bone Mineral Density: Results of a Stimulus Focussed Meta-Analysis
Source: PLoS One. 2016 Dec 1;11(12):e0166774. doi: 10.1371/journal.pone.0166774 (PMC5132247; doi:10.1371/journal.pone.0166774)
Supplement: S2 File — (DOC) [file pone.0166774.s002.doc]

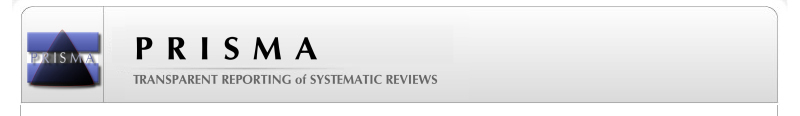
**PRISMA 2009 Flow Diagram**

**Screening**

**Included**

**Eligibility**

**Identification**

Records identified through database searching
(n =353)

Additional records identified through other sources
(n =5)

Records after duplicates removed
(n = 165)

Records screened
(n = 165)

Records excluded
(n = 143)

Full-text articles assessed for eligibility
(n = 22)

Full-text articles excluded
(n = 15)

Studies included in quantitative synthesis (meta-analysis)
(n = 7)
